# Smoothing the energy transfer pathway in quasi-2D perovskite films using methanesulfonate leads to highly efficient light-emitting devices

**DOI:** 10.1038/s41467-021-21522-8

**Published:** 2021-02-23

**Authors:** Lingmei Kong, Xiaoyu Zhang, Yunguo Li, Haoran Wang, Yuanzhi Jiang, Sheng Wang, Mengqing You, Chengxi Zhang, Ting Zhang, Stephen V. Kershaw, Weitao Zheng, Yingguo Yang, Qianqian Lin, Mingjian Yuan, Andrey L. Rogach, Xuyong Yang

**Affiliations:** 1 Key Laboratory of Advanced Display and System Applications of Ministry of Education, Shanghai University, Shanghai, 200072 People’s Republic of China; 2 College of Materials Science and Engineering, Jilin University, Changchun, 130012 People’s Republic of China; 3 CAS Key Laboratory of Crust-Mantle Materials and Environments, School of Earth and Space Sciences, University of Science and Technology of China, Hefei, 230026 People’s Republic of China; 4 Key Laboratory of Advanced Energy Materials Chemistry (Ministry of Education), Renewable Energy Conversion and Storage Center (RECAST), College of Chemistry, Nankai University, Tianjin, 300071 People’s Republic of China; 5 Department of Materials Science and Engineering, and Centre for Functional Photonics (CFP), City University of Hong Kong, Kowloon, Hong Kong SAR, People’s Republic of China; 6 Shanghai Synchrotron Radiation Facility (SSRF), Zhangjiang Lab, Shanghai Advanced Research Institute, Chinese Academy of Sciences, Shanghai, 201204 People’s Republic of China; 7 Key Lab of Artificial Micro- and Nano-Structures of Ministry of Education of China, School of Physics and Technology, Wuhan University, Wuhan, 430072 People’s Republic of China

**Keywords:** Materials for devices, Nanoscale devices, Lasers, LEDs and light sources

## Abstract

Quasi-two-dimensional (quasi-2D) Ruddlesden–Popper (RP) perovskites such as BA_2_Cs_*n*–1_Pb_*n*_Br_3*n*+1_ (BA = butylammonium, *n* > 1) are promising emitters, but their electroluminescence performance is limited by a severe non-radiative recombination during the energy transfer process. Here, we make use of methanesulfonate (MeS) that can interact with the spacer BA cations via strong hydrogen bonding interaction to reconstruct the quasi-2D perovskite structure, which increases the energy acceptor-to-donor ratio and enhances the energy transfer in perovskite films, thus improving the light emission efficiency. MeS additives also lower the defect density in RP perovskites, which is due to the elimination of uncoordinated Pb^2+^ by the electron-rich Lewis base MeS and the weakened adsorbate blocking effect. As a result, green light-emitting diodes fabricated using these quasi-2D RP perovskite films reach current efficiency of 63 cd A^−1^ and 20.5% external quantum efficiency, which are the best reported performance for devices based on quasi-2D perovskites so far.

## Abstract (web summary)

Owing to large exciton binding energy, quasi-2D perovskite is promising for light-emitting application, yet inhomogeneous phases distribution limits the potential. Here, the authors improve the performance by using MeS additive to regulate the phase distribution and to reduce defect density in the films.

## Introduction

Metal halide perovskites exhibit distinctive properties such as tunable bandgaps and narrow emission linewidths, which are combined with low cost and facile solution processability^1–10^. These useful characteristics have made them extremely promising candidates for light-emitting diode (LED) and display applications^11^. Tremendous progress has already been achieved in improving perovskite LEDs (PeLEDs) based on three-dimensional (3D) perovskite films^2,12–17^, but their overall electroluminescence (EL) performance still lags behind those of state-of-the-art organic LEDs and other solid-state lighting devices such as inorganic quantum-dot-based LEDs^11,18–20^. A slow electron–hole capture rate stemming from inherent small exciton binding (only tens of meV) and long diffusion length leads to inefficient radiative recombination in 3D perovskites^21–24^, going against high emission efficiency naturally.

Quasi-two-dimensional (quasi-2D) perovskites offer larger exciton binding energy (hundreds of meV) and lower ion mobility compared with 3D perovskites^23,25–28^, making them even more promising light-emitting materials. Quasi-2D perovskites with Ruddlesden–Popper (RP) structure have the general formula of L_2_A_*n*−1_M_*n*_X_3*n*+1_^23,24,29^, where L is a large-size organic spacer cation with low conductivity (“barrier”) such as butylammonium (BA), phenylethylammonium (PEA), naphthylmethylamine (NMA), etc., A is Cs, methylammonium (MA), or formamidinium (FA), M is a divalent metal cation such as Pb or Sn, X is a halide anion (Cl, Br, or I), and *n* is the number of MX_6_^4-^ sheets (which constitute a “quantum well”, QW) sandwiched between the organic spacers^25^. There are two key points that determine the emission efficiency of quasi-2D RP perovskites, namely the exciton energy transfer process and the deep defect state formation derived from the bandgap broadening^30–32^. The distribution of energy domains is inhomogeneous in quasi-2D perovskites due to the random stacking of low-dimensional structures with varying degrees of quantum and dielectric confinement^6,33,34^. The small-*n* phases (*n* ≤ 3) with wide-bandgap QWs are prone to form because of their low formation energy^35^, resulting in inefficient energy transfer processes from small-*n* phases to large-*n* phases. The defect states that result from lattice defects and the bandgap broadening due to the quantum confinement effect, act as charge-carrier traps during the energy transfer process, which lowers the emission efficiency.

Here, we seek to realize the full potential of quasi-2D perovskites for LEDs by smoothing the energy transfer pathway through structure reconstruction and defect reduction using methanesulfonate (MeS) additive. The strong hydrogen-bonding interaction between MeS and BA spacer cations effectively modulates the crystallization kinetics and leads to an increased ratio of large-*n* phase with narrow bandgap QWs, while the uncoordinated Pb^2+^ ions on the perovskite surface are simultaneously reduced due to the weakened adsorbate blocking effect caused by MeS addition, as well as the formation of Lewis adducts. Quasi-2D green PeLEDs fabricated following this strategy show current efficiency (CE) of 63 cd A^−1^ and up to 20.5% external quantum efficiency (EQE), making this device the best-performing quasi-2D perovskite LED reported in literature so far.

## Results

### Fabrication of MeS-treated BA_2_Cs_*n*−1_Pb_*n*_Br_3*n*+1_ perovskite films

**Fig. 1:**
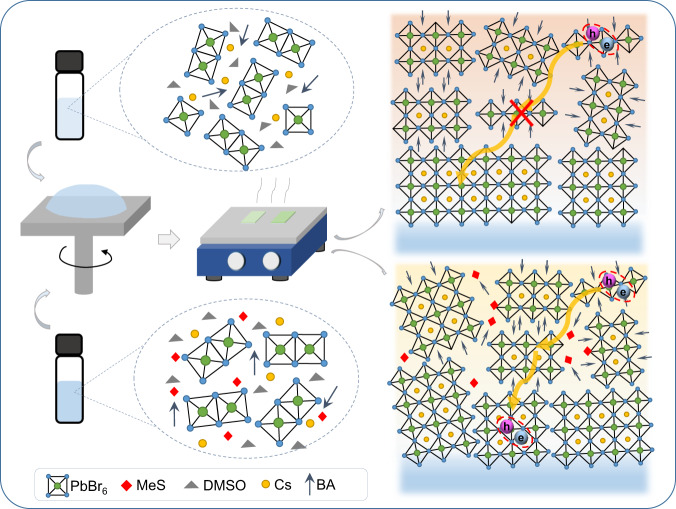
Effects of MeS on phase distribution of BA_2_Cs_*n*−1_Pb_*n*_Br_3*n*+1_ perovskite films. Schematic illustrations of the colloidal clusters’ structures in precursor solutions and phase distributions of *n* values in films after thermal annealing without (top pathway) and with (bottom pathway) addition of MeS, where different energy transfer pathways from small-*n* phases to large-*n* phases are presented. Crystal orientations in the perovskite films were verified by grazing-incidence wide-angle X-ray scattering (GIWAXS) measurements (see Supplementary Fig. 2).

The perovskite precursor solutions comprised of cesium bromide (CsBr), lead bromide (PbBr_2_), butylammonium bromide (BABr), poly(ethylene oxide) (PEO), and a varying ratio of cesium methanesulfonate (CsMeS) in dimethyl sulfoxide (DMSO) (see Methods for details). Here, the ratio of the added MeS is defined as the molar ratio of CsMeS-to-PbBr_2_ (denoted as “*x*%-MeS” further on). The MeS-treated quasi-2D perovskite films with a chemical formula of BA_2_Cs_*n*−1_Pb_*n*_Br_3*n*+1_ were prepared on indium tin oxide (ITO) substrates pre-coated with poly(9,9-dioctylfluorenyl-2,7-diyl)-co-(4,4′-(N-(4-sec-butylphenyl)diphenylamine) (TFB): poly(9-vinylcarbazole) (PVK) hole transport layer (HTL) and polyvinylpyrrolidone (PVP) interface modified layer by one-step solution coating of the precursor solutions, followed by thermal annealing under nitrogen atmosphere, as schematically illustrated in Fig. 1. The CsMeS molecule consists of two parts: a Cs^+^ cation and a MeS^−^ anion, whose molecular structure is shown in Supplementary Fig. 1. The introduction of MeS in precursor solution can control the crystallization growth kinetics of quasi-2D perovskite films resulting in enhanced exciton energy transfer from small-*n* phases to large-*n* phases, which will be discussed below in detail.

### Effect of MeS addition on growth of quasi-2D perovskite films

**Fig. 2:**
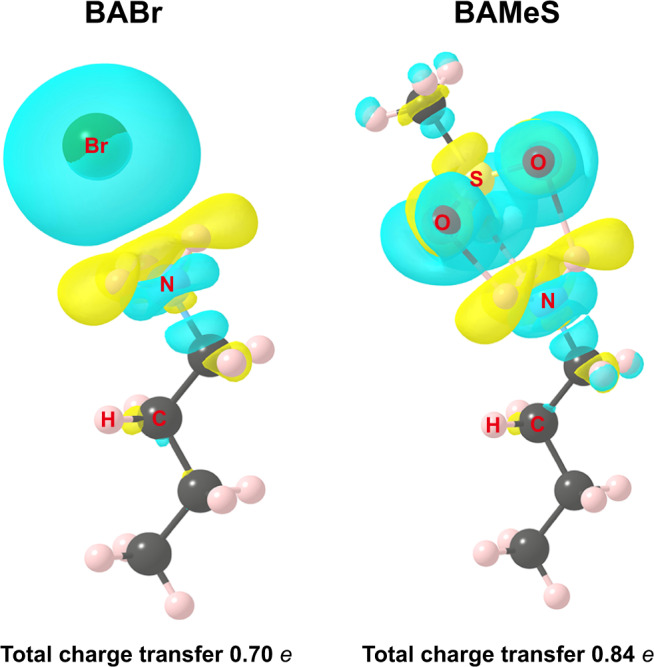
Hydrogen bonding calculations. Differential charge density plots (isosurface value of 0.0015 eV Å^−3^; cyan, charge accumulation; yellow, charge depletion) of BABr and BAMeS show that the bonding between BA and MeS induces more charge redistribution than that between BA and Br.

Density functional theory (DFT) calculations (see Methods) reveal that the spacer BA prefers to bind MeS via three hydrogen bonds rather than to bind Br anion. The following reaction was investigated by DFT simulations:

1 $${\mathrm{BABr}} + {\mathrm{CsMeS}} = {\mathrm{BAMeS}} + {\mathrm{CsBr}}$$

where BABr, CsMeS, and BAMeS were considered in the form of free molecules, and CsBr in the crystalline form of space group $Fm\bar 3m$. Their relaxed structures are shown in Supplementary Fig. 1. The calculated reaction free energy is −2.16 eV, indicating a strong driving force toward the formation of BAMeS. Indeed, the binding energy of BA and MeS was calculated to be 4.96 eV, which is much higher than both of BA and Br (4.00 eV), and Cs and MeS (4.39 eV). This suggests that MeS tends to bind BA via triple hydrogen bonding. The formation of hydrogen bonding incurs a charge redistribution of 0.84 e (electron) between BA and MeS, which is larger than that (0.70 e) between BA and Br (Fig. 2). Therefore, the strong affinity of the spacer cation for MeS fosters a higher probability for spacer-free nanosheet formation, and their stacking growth. This interaction is further confirmed by ^1^H nuclear magnetic resonance (NMR) spectra (Supplementary Fig. 3). The proton resonance signal of ammonium (NH_3_^+^) of BA (peak at δ = 7.65 ppm) shifts to low-field after incorporation of MeS (7.70 ppm). The variation in the chemical shift indicates the formation of hydrogen bonds between BA and MeS, which leads to the deshielding effect^36,37^. This enables us to increase the proportion of large-*n* phases in the overall perovskite phase distribution.

MeS molecules are homogeneously distributed over the quasi-2D perovskite films as shown in the energy-dispersive X-ray (EDX) mapping data (Supplementary Fig. 4). To address the influence of MeS on the perovskite phase distribution, measurements of the X-ray photoelectron spectra (XPS) that track the N signal (where BA is the only source of that atomic species and thus the signal stands for the phase distribution changes) along with decreasing the perovskite film thickness by increasing the Ar^+^ etching time were conducted. The gradual decrease in N content for both control and MeS-treated quasi-2D perovskite films were revealed (Supplementary Fig. 5), indicating that the multiple phases existing in quasi-2D perovskite films are arranged from small-*n* to large-*n* in the direction perpendicular to the substrate. The S signal (where MeS was the only source of that species) has a gradient content from high to low across the film from the top to the bottom (Supplementary Fig. 5b), resulting in an orderly phase arrangement in the vertical crystal spatial distribution.

Absorption spectra were measured to further analyze the effect of MeS on the phase distribution of quasi-2D perovskites. The control film produced without MeS treatment exhibits several exciton absorption peaks located at 403, 434, 467, and 516 nm (Supplementary Fig. 6), which correspond to phases with *n* = 1, 2, 3, and *n* ≥ 4, respectively^38–40^. The MeS-treated film exhibits weaker absorption peaks for small-*n* phases, and the 10%-MeS-treated film exhibits only one sharp exciton absorption peak (Supplementary Fig. 7a), suggesting a decrease in the content of small-*n* phases as the MeS content increased. As shown in the corresponding XRD patterns (Supplementary Fig. 7b, c), the appearance of the (002) plane^41^ for the *n* = 1 phase is hindered with increasing MeS content, consistent with the absorption analysis.

### Energy transfer in quasi-2D perovskite films

**Fig. 3:**
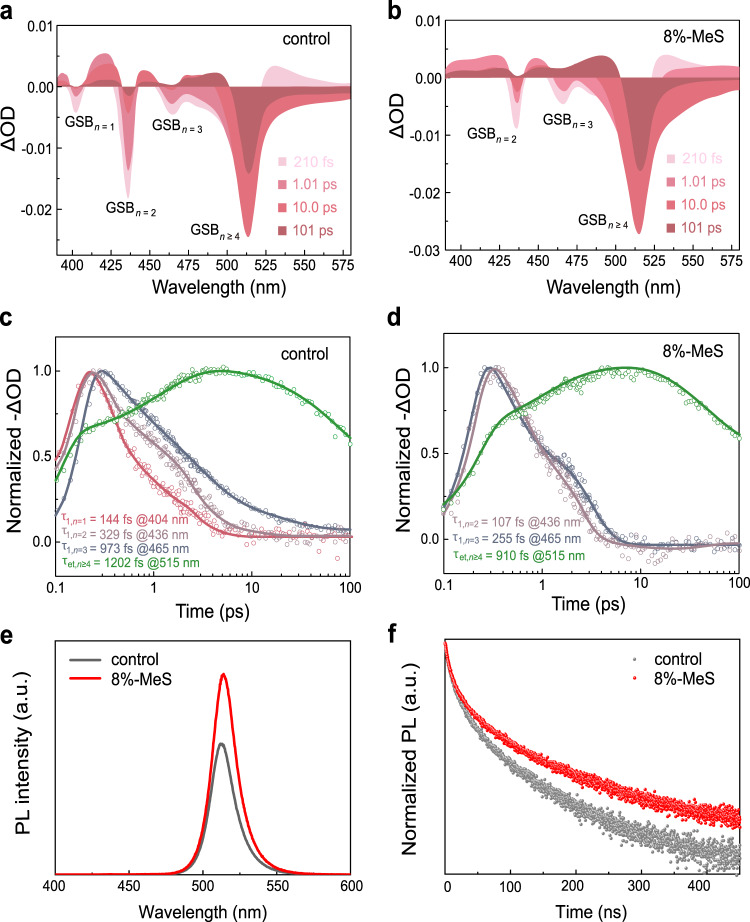
TA spectra and PL characteristics of quasi-2D perovskite films. **a**, **b** TA spectra at selected timescales. **c**, **d** TA kinetics traces probed at a different wavelength for the control (**a**, **c**) and MeS-treated perovskite films (**b**, **d**), respectively (excited at 365 nm). **e** Steady-state PL spectra and **f** TRPL decay of the control (emission wavelength: 512 nm) and MeS-treated perovskite film on glass substrates (emission wavelength: 514 nm) excited at 365 nm.

The exciton energy transfer in quasi-2D perovskite films was studied by transient absorption (TA) spectroscopy and steady-state photoluminescence (PL), and time-resolved photoluminescence (TRPL) spectroscopy. TA spectra shown in Fig. 3a–d reveal how the addition of MeS influences the dynamics of photo-generated carriers within quasi-2D perovskite films. The control perovskite film features four pronounced ground-state bleach (GSB) peaks at 404, 436, 465, and 513 nm corresponding to *n* = 1, 2, 3, and *n* ≥ 4 phases in the TA spectra at different selected delay times, respectively (Fig. 3a). The peak positions of these transitions are consistent with those observed in the steady-state absorption spectra (Supplementary Fig. 7a). Note that the exciton resonance at GSB_*n* = 2_ persists after a long excitation time (101 ps), suggesting that there are excitons accumulated in the *n* = 2 phase in particular, resulting from the incomplete energy transfer between the different phases^42^.

For the 8%-MeS-treated perovskite film, the exciton resonance at GSB_*n* = 1_ does not appear in the TA spectrum (Fig. 3b), indicating that the population fraction of the small-*n* phases was dramatically reduced. At a probe time of 101 ps, only the exciton bleach at GSB_*n* ≥ 4_ was observed in this sample. This is further confirmed by visualizing the same data as TA kinetics traces (Fig. 2c, d). The ultrafast decay component for the control film exhibits time constants (τ_1_) of 144 fs (*n* = 1), 329 fs (*n* = 2), and 973 fs (*n* = 3), which is closely matched with the formation (rise) time constant (τ_et_) of 1202 fs for GSB_*n* ≥ 4_ (Supplementary Table 1). In contrast, the decay kinetics for GSB_*n* ≥ 4_ peak of 8%-MeS-treated perovskite film shows a shorter formation time of 910 fs, demonstrating that an accelerated exciton energy transfer occurs inside the reconstructed phases enabling more efficient energy transfer from small-*n* phases to large-*n* phases.

The steady-state PL spectra and TRPL spectroscopy analysis are in good agreement with the results of TA kinetics. The 8%-MeS-treated perovskite film shows a much higher PL quantum yield (PLQY) of 73% than that of 47% for the control perovskite film (Fig. 3e and Supplementary Fig. 8). Such a dramatic improvement can be attributed to the improved energy transfer from small-*n* to large-*n* phases, ultimately enhancing the radiative recombination in the large-*n* phases. We also observed a slight redshift of the PL emission upon increasing the MeS content which is caused by the increased proportion of large-*n* phases^24^. TRPL spectroscopy shows an average recombination lifetime (τ_avg_) of 101 ns for 8%-MeS-treated films (Supplementary Table 2), which constitutes a 1.3-fold increase as compared to the control perovskite film (77 ns), corresponding to the enlarged perovskite crystal sizes (shift to higher *n*) after MeS treatment. For the control perovskite film, the PLQY was around 47% which, if the proportion of dark emitters is neglected, would imply almost equal radiative and non-radiative recombination rates. The fact that the PLQY increases to 73% with the MeS-treated perovskite, even though the decay rate is slower, indicates that the effective non-radiative rate must also have significantly slowed down (or the dark fraction of emitters has significantly dropped), which in any case would result in brighter emission (and indeed, both of these factors could have jointly contributed to the improved PLQY). In the further film measurements discussed below, we offer evidence that a significant contribution to the reduction in the non-radiative rate is the decrease of trap densities in MeS-treated perovskite.

**Fig. 4:**
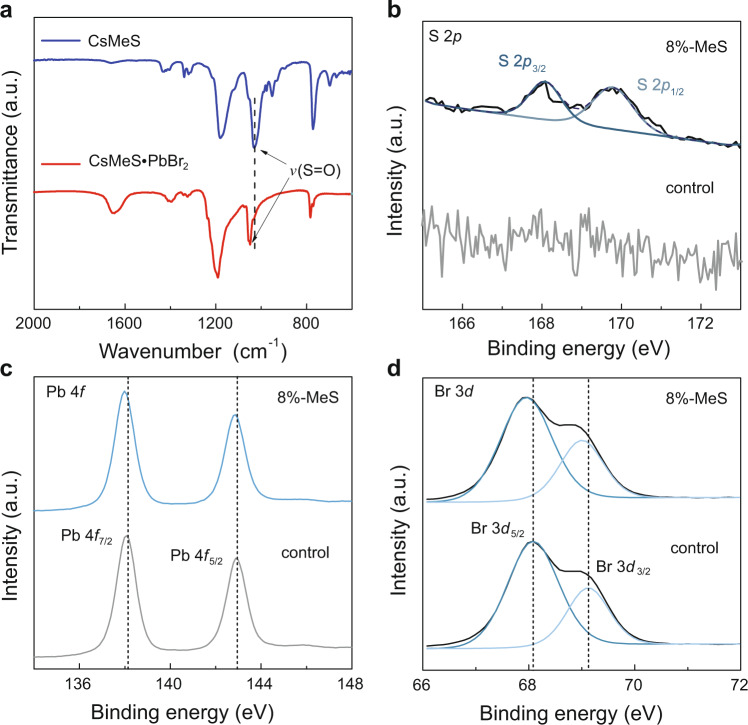
FTIR and XPS spectra. **a** FTIR spectra of powdered CsMeS and CsMeS·PbBr_2_, and high-resolution XPS spectra of the control and MeS-treated films for: **b** S 2*p*, **c** Pb 4*f*, and **d** Br 3*d*.

Apart from the structural reconstruction of perovskite films, the elimination (or partial elimination) of uncoordinated Pb^2+^ ions is yet another factor that ensures a more efficient exciton energy transfer pathway. Excitons trapped by uncoordinated Pb^2+^ are prone to recombine non-radiatively^43^. For the case of the samples discussed here, oxygen (O) atoms possessing lone pair electrons on the S = O groups of MeS could coordinate with the Pb-exposed surface through the formation of Lewis adducts^44^, favoring an efficient energy transfer into the emitting phases (as illustrated in Fig. 3f). Also, the reduction of small-*n* phases—which would appear to feature a greater abundance of non-radiative trap sites—should improve the radiative recombination^45^. Evidence of the coordination between MeS and Pb^2+^ is obtained from Fourier transform infrared (FTIR) and XPS (Fig. 4). The peak at 1029 cm^−1^ derived from the S=O stretching vibration (*ν* (S=O)) of the -SO_3_^−^ in FTIR spectra of powdered CsMeS^41^ shifts to a higher wavenumber of 1052 cm^−1^ in the presence of PbBr_2_, indicating the coordination of MeS and Pb^2+^ cations^46–48^. To further confirm whether this coordination exists in the MeS-treated perovskite films, we conducted XPS analysis of high-resolution S 2*p*, Pb 4*f*, and Br 3*d* chemical states. As shown in Fig. 4b, the peaks at 168.1 and 169.8 eV corresponding to respective S 2*p*_3/2_ and S 2*p*_1/2_ are observed in the MeS-treated perovskite film^49^. Figure 4c, d shows the XPS spectra for Pb and Br, respectively, where for the photoemission of Pb 4*f*, both Pb 4*f*_7/2_ (138.2 eV) and Pb 4*f*_5/2_ (142.9 eV), signals in the 8%-MeS-treated film shift to lower binding energy by around 0.2 eV. Such negative shifts of Pb 4*f* binding energy indicate the decrease of the cationic charge on the Pb ion since the S=O donates the lone electron pair on the oxygen atoms to the empty 6*p* orbital of Pb^2+^ cations^49^. The shift toward lower binding energy is also observed for the Br *3d* spectrum, which is caused by the disturbance due to the electron donation from MeS to Pb^2+^ through the formation of Lewis adducts, subsequently resulting also in the change of static interactions between Pb^2+^ and Br^−^ ions^50^. Besides, the strong affinity of BA with MeS reduces the chance of BA adsorption on the perovskite surface, and this weakens the blocking effect of BA on the growth of the perovskite. As the blocking effect of the adsorbate tends to induce defects in crystal growth^51–53^, the reduced BA adsorption caused by the presence of MeS may also contribute to alleviating defect formation in the perovskite films.

To support this hypothesis, the defect density was evaluated for hole-only devices with the structures of ITO/TFB:PVK/PVP/perovskite/MoO_3_/Al by performing space-charge-limited current (SCLC) measurements (Supplementary Fig. 9). The device current has a linear proportionality to the drive voltage up to the trap-filled limit (*V*_TFL_)^54–56^. It can be seen that the MeS-treated film presented a lower *V*_TFL_ (1.08 V) compared to those of the control film (1.45 V), demonstrating that the trap density in the perovskite film was reduced. Consequently, we extracted a trap density of *n*_traps_ = (5.05 ± 0.18) × 10^17^ cm^−3^ for the MeS-treated film, which is lower than that of the control film (*n*_traps_ = (9.87 ± 0.45) × 10^17^ cm^−3^). We further observed that, when the perovskite films were excited under different excitation powers, both the PLQYs of the control and MeS-treated films increased with the increase of excitation power, and the MeS-treated films showed remarkably higher PLQY values over the whole excitation range (Supplementary Fig. 10) due to the increased fraction of bimolecular recombination and trap-filling behavior^17^.

### Exciton energy transfer manipulation for efficient PeLEDs

**Fig. 5:**
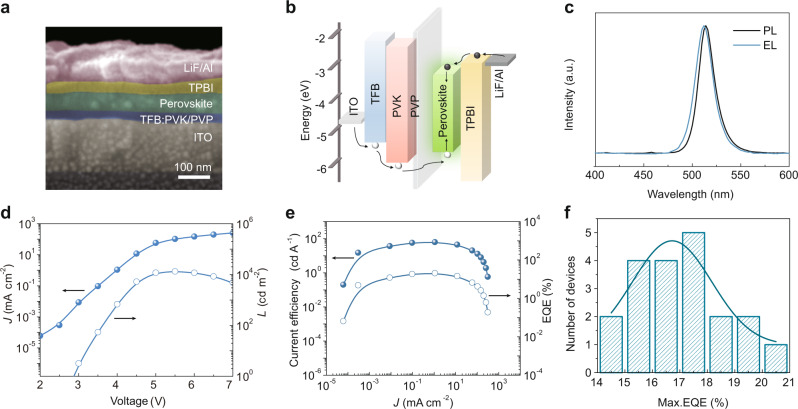
Device structure and performance of PeLEDs. **a** Cross-sectional SEM image of a PeLED with a structure of ITO/TFB:PVK (25 nm)/PVP (4 nm)/perovskite (50 nm)/TPBI (35 nm)/LiF (1 nm)/Al (100 nm), and **b** its schematic energy band diagram. Except for the perovskite layer which is directly measured, the energy level values for the other functional layers are taken from Lu et al.^11^. **c** Normalized PL spectrum of the 8%-MeS film, and EL spectrum of the PeLEDs at an applied voltage of 5.5 V. **d** *J–V–L*, **e** CE*–J–*EQE curves of the best-performing 8%-MeS-treated PeLED, and **f** histogram of the maximum EQEs measured on 20 such devices.

Green PeLEDs with structures of ITO/TFB:PVK/control perovskite/1,3,5-tris(1-phenyl-1H-benzimidazol-2-yl)benzene (TPBI)/lithium fluoride (LiF)/aluminum (Al) (Device A), ITO/TFB:PVK/MeS-treated perovskite/TPBI/LiF/Al (Device B), ITO/TFB:PVK/PVP/MeS-treated perovskite/TPBI/LiF/Al (Device C), and ITO/TFB:PVK/PVP/control perovskite/TPBI/LiF/Al (Device D) have been fabricated and compared. In the cross-sectional scanning electron microscopy (SEM) image (Fig. 5a), each of the functional layers in Device C (except for the ultrathin, ~4 nm PVP film) can be clearly seen. The device energy band diagram is plotted in Fig. 5b, based on energy level values for this perovskite film as obtained by ultraviolet photoelectron spectroscopy (UPS) coupled with optical measurements (Supplementary Fig. 11). Note that the presence of an ultrathin layer of PVP on top of the TFB:PVK mixed HTL plays an important role in improving film quality and ensuing better device performance (Supplementary Fig. 12). It not only enables the deposition of smooth and dense perovskite films due to the significantly improved HTL surface wettability (Supplementary Fig. 13), but also suppresses the emission quenching by suppressing charge transfer at the perovskite/HTL interface^57,58^ (Supplementary Fig. 14). In the absence of PVP (in both cases), Device B shows an almost two-fold performance enhancement compared to the Device A (13.14% EQE & 10,140 cd m^−2^ luminance vs. 6.37% EQE & 6094 cd m^−2^ luminance, respectively. Supplementary Fig. 15 and Supplementary Table 3).

Figure 5c–f shows the performance characteristics of our best green PeLED with MeS-treated perovskite and PVP interlayer (Device C). The EL spectrum matches well with the PL spectrum (Fig. 5c), and the EL emission peak (at 512 nm) is only slightly blueshifted and broadened. The blueshift most probably results from free carrier emission, as already demonstrated in several perovskite systems^59–61^; and the broadened EL can be attributed to the electric-field-induced Stark effect^2,62^. From the current density–luminance–voltage (*J–V–L*) curves (Fig. 5d), we can see that the current density rapidly increased after reaching the turn-on voltage (~2.8 V) due to efficient carrier injection into the perovskite emission layer. As the current density continued to increase, the device had a peak luminance of 13,400 cd m^−2^ at a low applied voltage of 5.5 V, and a high CE of 63 cd A^−1^ corresponding to an EQE of 20.5% at a current density of ~1 mA cm^−2^, which is, to the best of our knowledge, a record efficiency in quasi-2D PeLEDs for all colors (Supplementary Table 4 compares our results with recently reported values in the literature). A histogram of the maximum EQE values for 20 devices (Fig. 5f) shows an average EQE of 17.1% with a relative standard deviation of 10%, indicating good device-to-device reproducibility. Furthermore, due to the reduced ion migration pathways in the MeS-treated perovskite films, the device stability becomes remarkably improved as well, so that the Device C shows a two-fold operational lifetime enhancement compared with the Device D (Supplementary Fig. 16).

## Discussion

We achieved a record efficiency (CE of 63 cd A^−1^ and EQE of 20.5%) for quasi-2D BA_2_Cs_*n*−1_Pb_*n*_Br_3*n*+1_ PeLEDs by introducing MeS into the perovskite precursor solutions to reconstruct the perovskite phase distribution and reduce uncoordinated Pb^2+^ ions, and thus enhance the emission efficiency of perovskite films derived from these solutions. The proportion of large-*n* phases with narrow bandgap has increased in the distribution phases of different *n* values of quasi-2D perovskites due to hydrogen-bonding interaction between -SO_3_^−^ group of MeS and BA spacer; and the surface uncoordinated Pb^2+^ ions on the perovskites were simultaneously reduced benefiting from the electron-rich Lewis base nature of MeS. Besides, the blocking effect of BA which tends to induce defects during crystal growth could be reduced because of the interaction between BA and MeS. This work offers a promising approach for fabricating next-generation high-performance PeLEDs based on quasi-2D perovskites.

## Methods

### Materials

CsBr (99.999%, metal basis) and PbBr_2_ (99.999%, metal basis) were purchased from Alfa Aesar. BABr was purchased from Xi’an Polymer Light Technology Corp. CsMeS, PVK, PVP, PEO, and DMSO (99.9%) were purchased from Sigma-Aldrich. TFB, TPBI, and LiF were purchased from Luminescence Technology Corp. Absolute ethyl alcohol and toluene were purchased from Sinopharm Chemical Reagent Co., Ltd.

### Perovskite precursor solutions

PbBr_2_, CsBr, and BABr were dissolved in anhydrous DMSO with the ratio of 1:1.2:0.4, and PEO (3.33 mg ml^−1^) was added to improve the morphology of the resulting film^63–65^. CsMeS was added into the precursor solution, with its molar ratios to PbBr_2_ being 6%, 8%, and 10%, respectively. The mixture was stirred at 60 °C overnight and filtered through a 0.45 μm polytetrafluoroethylene membrane before using.

### Device Fabrication

ITO substrates were sonicated in sequence with detergent, deionized water, acetone, and isopropyl alcohol for 20 min, respectively, and then dried under nitrogen flow. TFB and PVK were mixed in a weight ratio of 4:6 and dissolved in chlorobenzene (10 mg ml^−1^). A TFB:PVK layer was spin-coated onto oxygen-plasma-treated ITO substrates at 4000 rpm for 40 s, followed by annealing at 150 °C for 20 min. PVP (1.5 mg ml^−1^ in absolute ethyl alcohol) was spin-coated on top of the TFB:PVK layer. For the perovskite film, the precursor solution was spin-coated at 4000 rpm for 50 s—after spin coating for 40 s, 100 µl toluene antisolvent was dropped onto the film and spin coating continued for a further 10 s—followed by baking at 80 °C for 10 min. After that, TPBI (40 nm), LiF (1 nm), and Al electrode (100 nm) layers were sequentially deposited by thermal evaporation under a vacuum of ~4 × 10^−4^ Pa.

### Characterization

XRD patterns were collected on a Bruker D8 Advance diffractometer with Cu Kα radiation as the X-ray source, ranging from 5° to 60° at a scanning rate of 6° min^−1^. UV-Vis spectra were recorded on a Perkin Elmer Lambda 950 UV-Vis-NIR spectrometer. TA measurements were carried out on a Helios pump-probe system (Ultrafast Systems LLC) with a 365-nm laser (0.17 mW). XPS and UPS spectra were acquired using a Thermo Scientific Escalab 250Xi. He (I) ultraviolet radiation source (21.22 eV) from a He discharge lamp was used in the UPS measurements. The work functions (WFs) and the highest occupied molecular orbital (HOMO) regions were extracted from the UPS spectra using the equations WF = 21.22 *−* *E*_cutoff_ and HOMO = WF + *E*_onset_. The *E*_cutoff_ and the *E*_onset_ were determined by the intercepts of the tangents of the peaks with the extrapolated baselines, as depicted in the relevant plots. Steady-state PL spectra and TRPL were recorded on an Edinburgh FLS920 PL spectrometer at an excitation wavelength of 365 nm. ^1^H NMR spectra were recorded on a Bruker Advanced II (400 MHz) spectrometer at room temperature. The grazing-incidence wide-angle X-ray scattering (GIWAXS) data were obtained at beamline BL14B1 of the Shanghai Synchrotron Radiation Facility (SSRF), China. A monochromatic beam of λ = 0.6887 Å was used, and the incident angle was 0.1°. The EL characteristics of the PeLEDs were collected on a system comprised of a PR-670 Spectra Scan Spectroradiometer coupled with a Keithley 2400 sourcemeter. The EQEs were calculated from the luminance, the current density, and the EL emission spectra of devices. All the device characterization tests were carried out on unencapsulated PeLEDs at room temperature in ambient air, except that the operational stability test was carried in an N_2_-filled glovebox.

### DFT simulations

The reaction Gibbs free energy Δ*G* for the reaction, CsMeS + BABr = BAMeS + CsBr, was approximated by the change of ground-state energy at 0 K: $\Delta G \approx E_{{\mathrm{BAMeS}}} + E_{{\mathrm{CsBr}}} - E_{{\mathrm{BABr}}} - E_{{\mathrm{CsMeS}}}$. The ground-state energy was calculated using the Vienna Ab Initio Simulations Package (VASP) and projected augmented wave (PAW) method with 1*s*^1^, 2*s*^2^2*p*^2^, 2*s*^2^2*p*^3^, 2*s*^2^2*p*^4^, 3*s*^2^3*p*^4^, 4*s*^2^4*p*^5^, 5*s*^2^5*p*^6^6*s*^1^, and 6*s*^2^6*p*^2^ as valence electrons for H, C, N, O, S, Br, Cs, and Pb, respectively. The exchange-correlation interaction was treated with the generalized gradient approximation (GGA) parameterized by a Perdew, Burke, and Ernzerhof (PBE) functional. The Brillouin zone was sampled by using a Gamma point for molecules and a Gamma-cantered k-mesh of 9 × 9 × 9 for CsBr. The cutoff energy of the plane-wave basis was chosen to converge the energy to 1 meV atom^−1^. The force was converged to less than 0.01 eV Å^−1^ for all structure relaxations. The calculated ground-state energies are −90.935, −46.738, −133.728, and −6.107 eV for BABr, CsMeS, BAMeS, and CsBr, respectively. The reaction free energy is therefore −2.16 eV.

## Supplementary information

Supplementary information.

## Data Availability

The data that support the plots within this paper and the other findings of this study are available from the corresponding authors upon reasonable request.

## References

[1] Liu Y (2019). Efficient blue light-emitting diodes based on quantum-confined bromide perovskite nanostructures. Nat. Photonics.

[2] Wang H (2019). Trifluoroacetate induced small-grained CsPbBr_3_ perovskite films result in efficient and stable light emitting devices. Nat. Commun..

[3] Kumar S (2017). Ultrapure green light-emitting diodes using two-dimensional formamidinium perovskites: achieving recommendation 2020 color coordinates. Nano Lett..

[4] Wang H (2020). Perovskite-molecule composite thin films for efficient and stable light-emitting diodes. Nat. Commun..

[5] Shi Z (2019). Uncovering the mechanism behind the improved stability of 2D organic-inorganic hybrid perovskites. Small.

[6] Deng S (2020). Long-range exciton transport and slow annihilation in two-dimensional hybrid perovskites. Nat. Commun..

[7] Adjokatse S, Fang H-H, Loi MA (2017). Broadly tunable metal halide perovskites for solid-state light-emission applications. Mater. Today.

[8] Lu J-H, Yu Y-L, Chuang S-R, Yeh C-H, Chen C-P (2016). High-performance, semitransparent, easily tunable vivid colorful perovskite photovoltaics featuring Ag/ITO/Ag microcavity structures. J. Phys. Chem. C.

[9] Wang Q (2019). Efficient sky-blue perovskite light-emitting diodes via photoluminescence enhancement. Nat. Commun..

[10] Jiang Y (2019). Spectra stable blue perovskite light-emitting diodes. Nat. Commun..

[11] Lu M (2019). Metal halide perovskite light‐emitting devices: promising technology for next‐generation displays. Adv. Funct. Mater..

[12] Zhao L, Lee KM, Roh K, Khan SUZ, Rand BP (2019). Improved outcoupling efficiency and stability of perovskite light‐emitting diodes using thin emitting layers. Adv. Mater..

[13] Yuan Z (2019). Unveiling the synergistic effect of precursor stoichiometry and interfacial reactions for perovskite light-emitting diodes. Nat. Commun..

[14] Cao Y (2018). Perovskite light-emitting diodes based on spontaneously formed submicrometre-scale structures. Nature.

[15] Xu W (2019). Rational molecular passivation for high-performance perovskite light-emitting diodes. Nat. Photonics.

[16] Lin K (2018). Perovskite light-emitting diodes with external quantum efficiency exceeding 20 percent. Nature.

[17] Wu T (2020). High-performance perovskite light-emitting diode with enhanced operational stability using lithium halide passivation. Angew. Chem. Int. Ed..

[18] Shen H (2019). Visible quantum dot light-emitting diodes with simultaneous high brightness and efficiency. Nat. Photonics.

[19] Song J (2019). Over 30% external quantum efficiency light‐emitting diodes by engineering quantum dot‐assisted energy level match for hole transport layer. Adv. Funct. Mater..

[20] Rajamalli P (2016). A new molecular design based on thermally activated delayed fluorescence for highly efficient organic light emitting diodes. J. Am. Chem. Soc..

[21] Tan Z-K (2014). Bright light-emitting diodes based on organometal halide perovskite. Nat. Nanotechnol..

[22] Xiao Z (2017). Efficient perovskite light-emitting diodes featuring nanometre-sized crystallites. Nat. Photonics.

[23] Liu T (2019). Tailoring vertical phase distribution of quasi-two-dimensional perovskite films via surface modification of hole-transporting layer. Nat. Commun..

[24] Zhou N (2017). Exploration of crystallization kinetics in quasi two-dimensional perovskite and high performance solar cells. J. Am. Chem. Soc..

[25] Cheng L (2019). Multiple‐quantum‐well perovskites for high‐performance light‐emitting diodes. Adv. Mater..

[26] Lin Y (2017). Suppressed ion migration in low-dimensional perovskites. ACS Energy Lett..

[27] Li C (2019). Understanding the improvement in the stability of a self-assembled multiple-quantum well perovskite light-emitting diode. J. Phys. Chem. Lett..

[28] Yang X (2018). Efficient green light-emitting diodes based on quasi-two-dimensional composition and phase engineered perovskite with surface passivation. Nat. Commun..

[29] Ban M (2018). Solution-processed perovskite light emitting diodes with efficiency exceeding 15% through additive-controlled nanostructure tailoring. Nat. Commun..

[30] Leng M (2018). Surface passivation of bismuth-based perovskite variant quantum dots to achieve efficient blue emission. Nano Lett..

[31] Yin W-J, Yang J-H, Kang J, Yan Y, Wei S-H (2015). Halide perovskite materials for solar cells: a theoretical review. J. Mater. Chem. A.

[32] Kim J, Chung C-H, Hong K-H (2016). Understanding of the formation of shallow level defects from the intrinsic defects of lead tri-halide perovskites. Phys. Chem. Chem. Phys..

[33] Sapori D (2016). Quantum confinement and dielectric profiles of colloidal nanoplatelets of halide inorganic and hybrid organic-inorganic perovskites. Nanoscale.

[34] Xia P (2019). A pre-solution mixing precursor method for improving crystallization quality of perovskite film and electroluminescent performance of perovskite light-emitting diodes. Nanoscale.

[35] Quan LN (2016). Ligand-stabilized reduced-dimensionality perovskites. J. Am. Chen. Soc..

[36] Du J (2018). Ionic liquid‐assisted improvements in the thermal stability of CH_3_NH_3_PbI_3_ perovskite photovoltaics. Phys. Status Solidi RRL.

[37] Yuan S (2019). Optimization of low‐dimensional components of quasi‐2D perovskite films for deep‐blue light‐emitting diodes. Adv. Mater..

[38] Quan LN (2017). Tailoring the energy landscape in quasi-2D halide perovskites enables efficient green-light emission. Nano Lett..

[39] Yuan M (2016). Perovskite energy funnels for efficient light-emitting diodes. Nat. Nanotechnol..

[40] Liu J, Leng J, Wu K, Zhang J, Jin S (2017). Observation of internal photoinduced electron and hole separation in hybrid two-dimensional perovskite films. J. Am. Chem. Soc..

[41] Cohen B-E, Wierzbowska M, Etgar L (2017). High efficiency quasi 2D lead bromide perovskite solar cells using various barrier molecules. Sustain. Energy Fuels.

[42] Lei L (2020). Effcient energy funneling in quasi-2D perovskites: from light emission to lasing. Adv. Mater..

[43] Cho H (2015). Overcoming the electroluminescence efficiency limitations of perovskite light-emitting diodes. Science.

[44] Ren Z (2019). Hole transport bilayer structure for quasi‐2D perovskite based blue light‐emitting diodes with high brightness and good spectral stability. Adv. Funct. Mater..

[45] Xing G (2017). Transcending the slow bimolecular recombination in lead-halide perovskites for electroluminescence. Nat. Commun..

[46] Zhou W (2019). Zwitterion coordination induced highly orientational order of CH_3_NH_3_PbI_3_ perovskite film delivers a high open circuit voltage exceeding 1.2 V. Adv. Funct. Mater..

[47] Wang Q (2017). Stabilizing the α-phase of CsPbI_3_ perovskite by sulfobetaine zwitterions in one-step spin-coating films. Joule.

[48] Deepa M, Sharma N, Agnihotry SA, Chandra R (2002). FTIR investigations on ion-ion interactions in liquid and gel polymeric electrolytes: LiCF_3_SO_3_-PC-PMMA. J. Mater. Sci..

[49] Han MG, Im SS (2000). X-ray photoelectron spectroscopy study of electrically conducting polyaniline/polyimide blends. Polymer.

[50] Li B (2018). Anchoring fullerene onto perovskite film via grafting pyridine toward enhanced electron transport in high-efficiency solar cells. ACS Appl. Mater. Interfaces.

[51] Chen D, Gallagher MJ, Sarid D (1994). Scanning tunneling microscopy study of the adsorption of C60 molecules on Si (100)‐(2 × 1) surfaces. J. Vac. Sci. Technol. B.

[52] Taubert A, Kübel C, Martin DC (2003). Polymer-induced microstructure variation in zinc oxide crystals precipitated from aqueous solution. J. Phys. Chem. B.

[53] Duan C (2019). Engineering new defects in MIL-100 (Fe) via a mixed-ligand approach to effect enhanced volatile organic compound adsorption capacity. Ind. Eng. Chem. Res..

[54] Shen X (2019). Zn-alloyed CsPbI_3_ nanocrystals for highly efficient perovskite light-emitting devices. Nano Lett..

[55] Wu X (2020). Stable triple cation perovskite precursor for highly efficient perovskite solar cells enabled by interaction with 18C6 stabilizer. Adv. Funct. Mater..

[56] Wang H (2020). Molecule‐induced p‐doping in perovskite nanocrystals enables efficient color‐saturated red light‐emitting diodes. Small.

[57] Zhang X (2016). Enhancing the brightness of cesium lead halide perovskite nanocrystal based green light-emitting devices through the interface engineering with perfluorinated ionomer. Nano Lett..

[58] Pal BN (2012). ‘Giant’ CdSe/CdS core/shell nanocrystal quantum dots as efficient electroluminescent materials: strong influence of shell thickness on light-emitting diode performance. Nano Lett..

[59] Zhang L (2017). Ultra-bright and highly efficient inorganic based perovskite light-emitting diodes. Nat. Commun..

[60] Palma AL (2016). Mesoscopic perovskite light-emitting diodes. ACS Appl. Mater. Interfaces.

[61] D’Innocenzo V (2014). Excitons versus free charges in organo-lead tri-halide perovskites. Nat. Commun..

[62] Yang X (2015). Electroluminescence efficiency enhancement in quantum dot light‐emitting diodes by embedding a silver nanoisland layer. Adv. Opt. Mater..

[63] Jeong B (2018). All-inorganic CsPbI_3_ perovskite phase-stabilized by poly (ethylene oxide) for red-light-emitting diodes. Adv. Funct. Mater..

[64] Tian Y (2018). Highly efficient spectrally stable red perovskite light-emitting diodes. Adv. Mater..

[65] Wu C (2017). Improved performance and stability of all-inorganic perovskite light-emitting diodes by antisolvent vapor treatment. Adv. Funct. Mater..

