# Supplementary material for: Smoothing the energy transfer pathway in quasi-2D perovskite films using methanesulfonate leads to highly efficient light-emitting devices
Source: Nat Commun. 2021 Feb 23;12:1246. doi: 10.1038/s41467-021-21522-8 (PMC7902836; doi:10.1038/s41467-021-21522-8)
Supplement: Supplementary file 1 — Supplementary information. [file 41467_2021_21522_MOESM1_ESM.pdf]

Supplementary Information for

Smoothing the energy transfer pathway in quasi-2D perovskite films using methanesulfonate leads to highly efficient light-emitting devices

Kong et al.

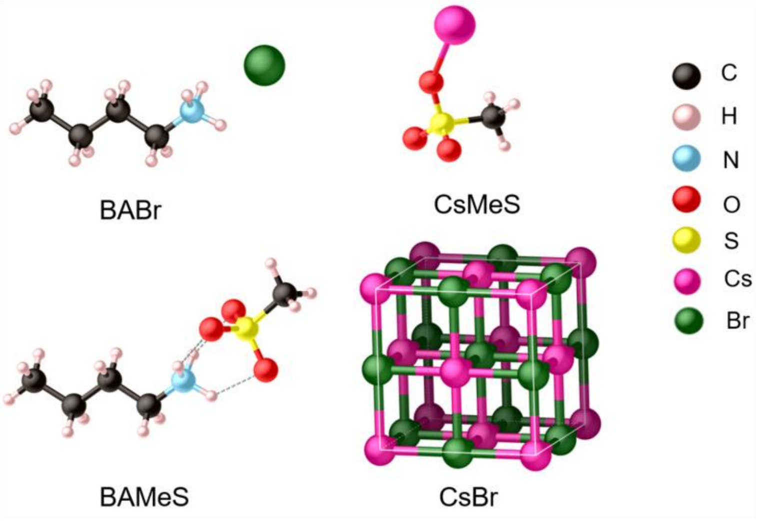

Supplementary Figure 1 | Molecular structures of BABr, CsMeS, BAMeS and crystal structure of CsBr. BA prefers to bind MeS *via* three hydrogen bonds according to the equation:

BABr + CsMeS = BAMeS + CsBr

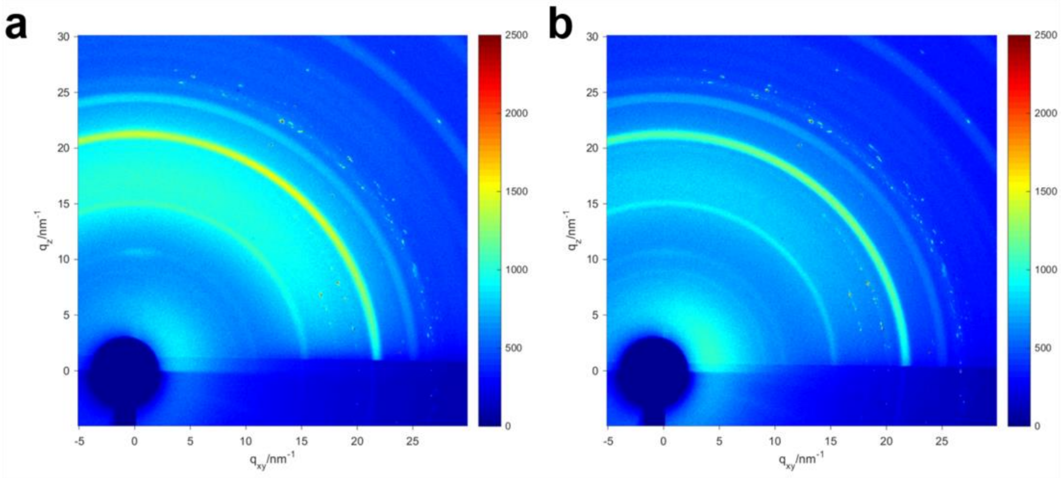

Supplementary Figure 2 | GIWAXS patterns of quasi-2D perovskite films. GIWAXS patterns of (a) control and (b) MeS-treated quasi-2D perovskite films. The MeS-treated perovskite film shows uniform intensities along the Debye-Scherrer ring compared to that of the control perovskite film, indicating considerably random crystal orientation in the MeS-treated film.

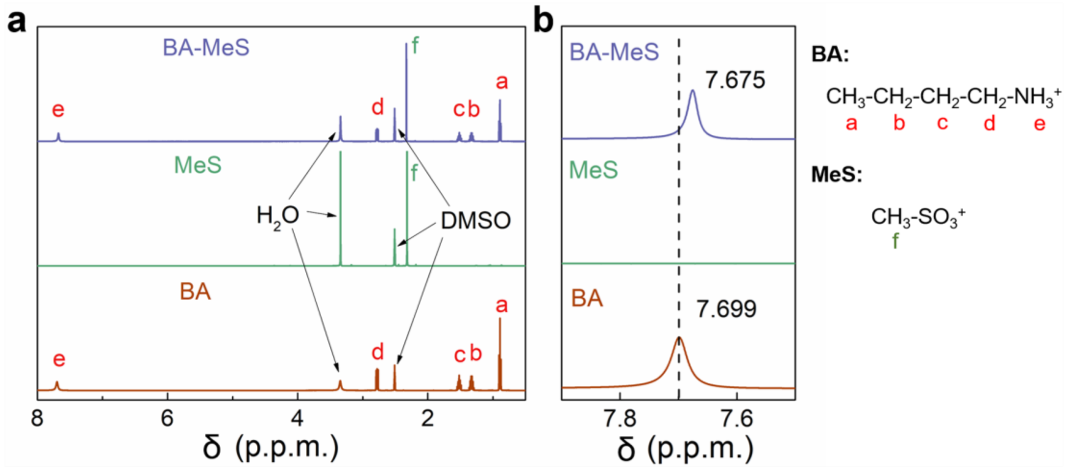

Supplementary Figure 3 | ^{1}H NMR spectra of BA, MeS and BA-MeS in deuterated DMSO solution. (a) Proton resonance signals of BA, MeS and BA-MeS (mole ratio: 1:1) in deuterated DMSO. (b) Zoom-in region of NH^{3+} proton resonance signal.

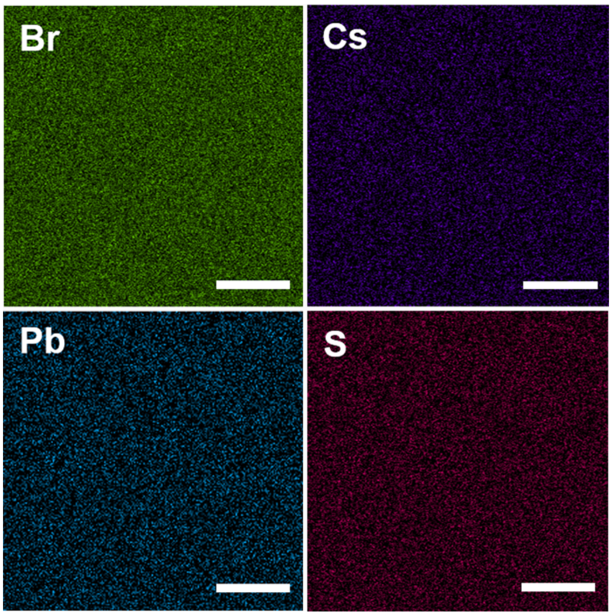

Supplementary Figure 4 | EDX mapping of the MeS-treated perovskite films. EDX mapping showing the homogeneous distribution of Br, Cs, Pb, and S elements in MeS-treated perovskite films. All scale bars are 2.5 μm.

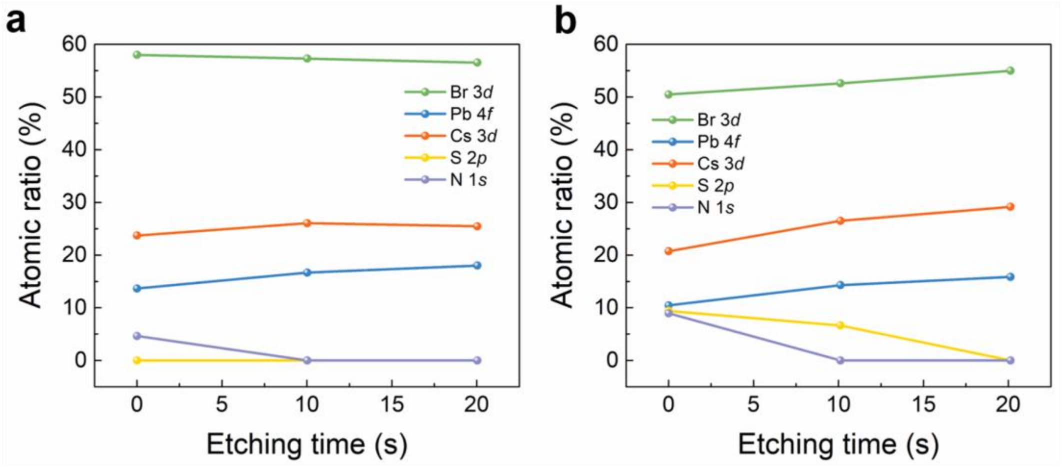

Supplementary Figure 5 | Atomic ratios of constituting elements before and after etching with Ar^{+} ions for (a) control and (b) MeS-treated perovskite films. It is revealed that the multiple phases existing in quasi-2D perovskite are arranged from small-n to large-n in the direction perpendicular to the substrate and the MeS in perovskite has a gradient content from high to low across the film from the top to bottom.

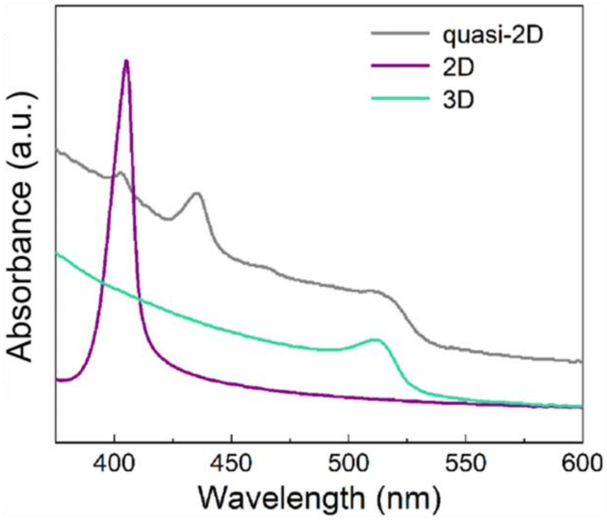

Supplementary Figure 6 | Absorption spectra of quasi-2D perovskite films. Absorption spectra of quasi-2D $BA_2Cs_{n-1}Pb_nBr_{3n+1}$, 2D BA_2PbBr_4 and 3D $CsPbBr_3$ films. As shown, quasi-2D perovskite films contain four phases in which includes 2D and 3D phases.

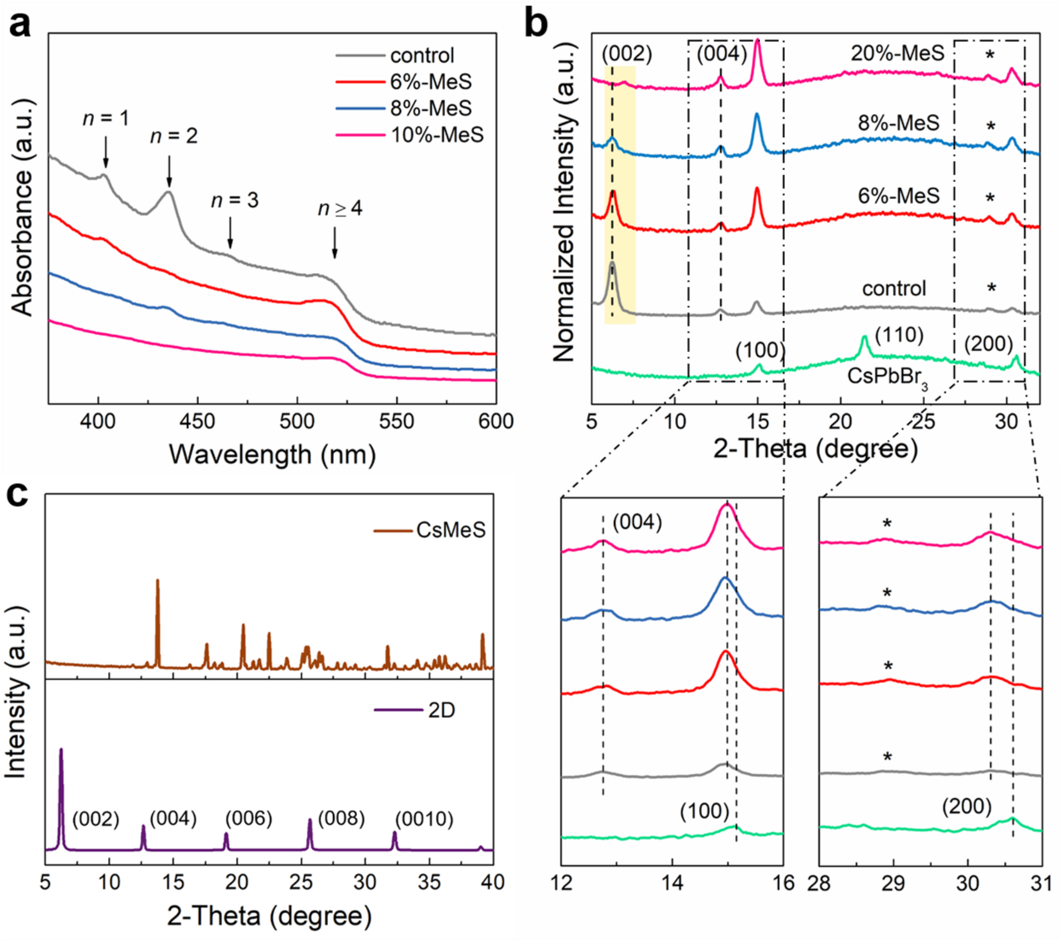

Supplementary Figure 7 | Absorption spectra and XRD patterns of perovskite films. (a) Absorption spectra of the control and *x*%-MeS treated quasi-2D perovskite films. (b) XRD patterns of $CsPbBr_3$ film and *x*%-MeS treated quasi-2D perovskite films; the dashed boxes indicate the corresponding zoom-in region. The peaks marked with * represent the peaks corresponding to quasi-2D perovskite. (c) XRD patterns of CsMeS and 2D BA_2PbBr_4 films.

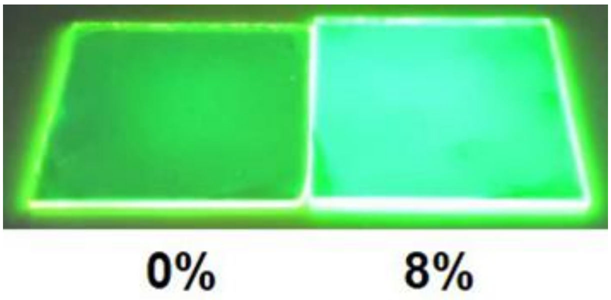

Supplementary Figure 8 | Photoluminescence image of quasi-2D perovskite films. Photoluminescence image of the control and MeS-treated perovskite films under ultraviolet lamp excitation (365 nm). It can be observed that the MeS-treated perovskite films show brighter green emission than the control film.

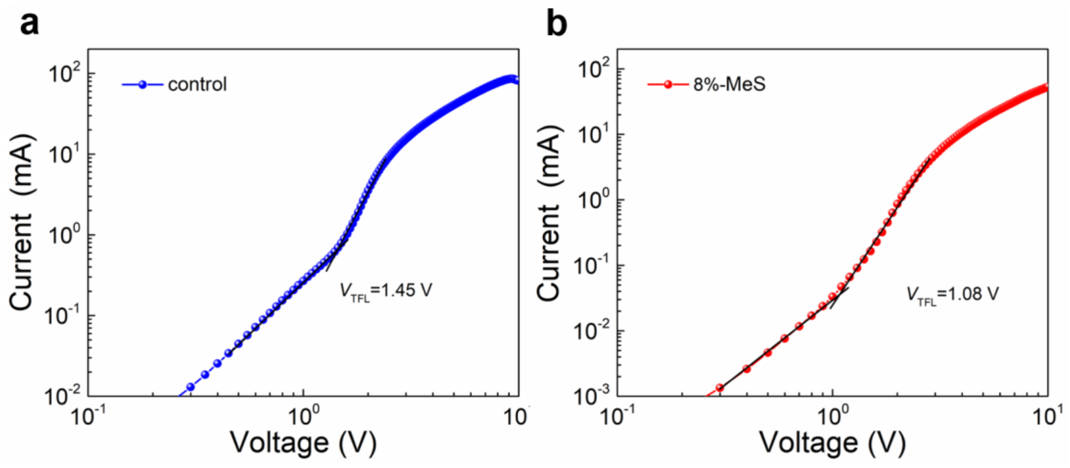

Supplementary Figure 9 | SCLC measurements for the perovskite films. Current-voltage characteristics of devices with (a) ITO/TFB:PVK/PVP/control perovskite/MoO_3/Al and (b) ITO/TFB:PVK/PVP/MeS-treated perovskite/MoO_3/Al configurations utilized for estimating the trap density in perovskite films. The defect density is calculated according to the equation: $n_{traps} = \frac{2\varepsilon\varepsilon_0 V_{\mathrm{TFL}}}{eL^2}$, where ε and ε_0 are the relative dielectric constant of the perovskite[1] and the vacuum permittivity, respectively, V_{TFL} is the trap-filled limit voltage, e is the elementary electronic charge, and L is the thickness of the perovskite film.

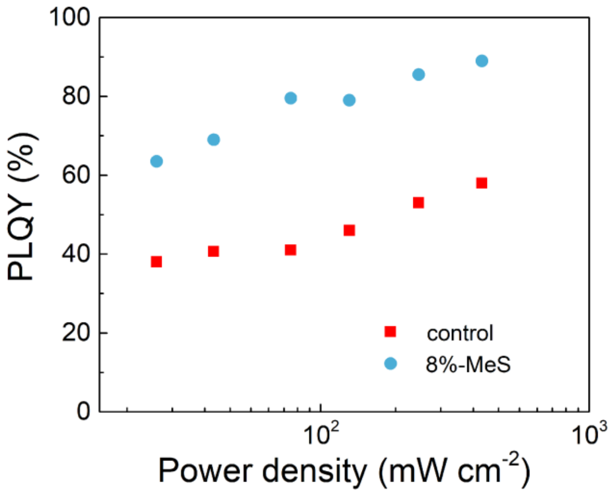

Supplementary Figure 10 | Power-density-dependent PLQYs of perovskite films. As shown, both the PLQYs of the control and MeS-treated films increased with the increase of excitation power, due to the increased fraction of bimolecular recombination and trap-filling behavior. While the MeS-treated films presented remarkably higher PLQY values over the whole excitation range.

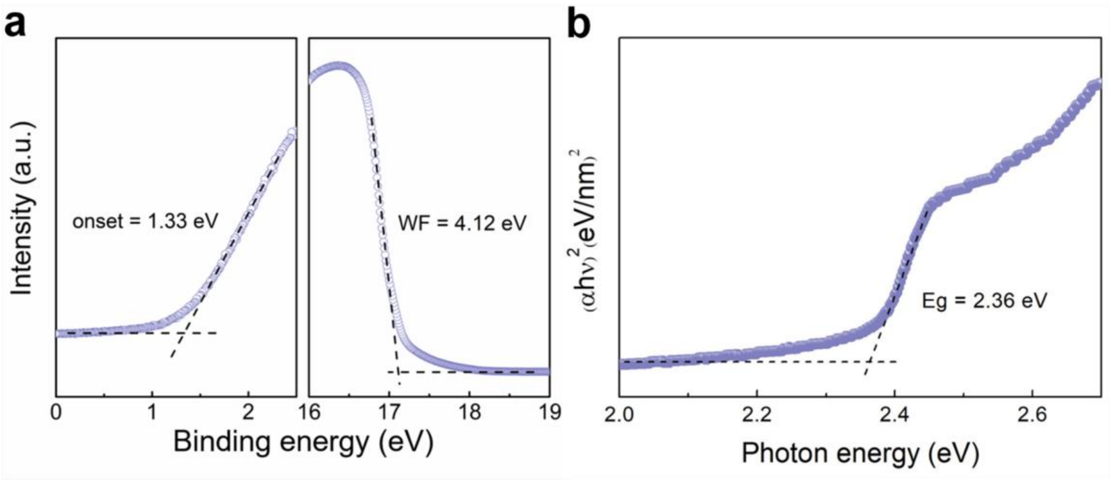

Supplementary Figure 11 | UPS spectra and Tauc plots of perovskite films. (a) UPS spectra of valence-band edge regions and high binding energy secondary-electron cutoffs of 8%-MeS treated perovskite film. (b) Tauc plots of 8%-MeS treated perovskite film.

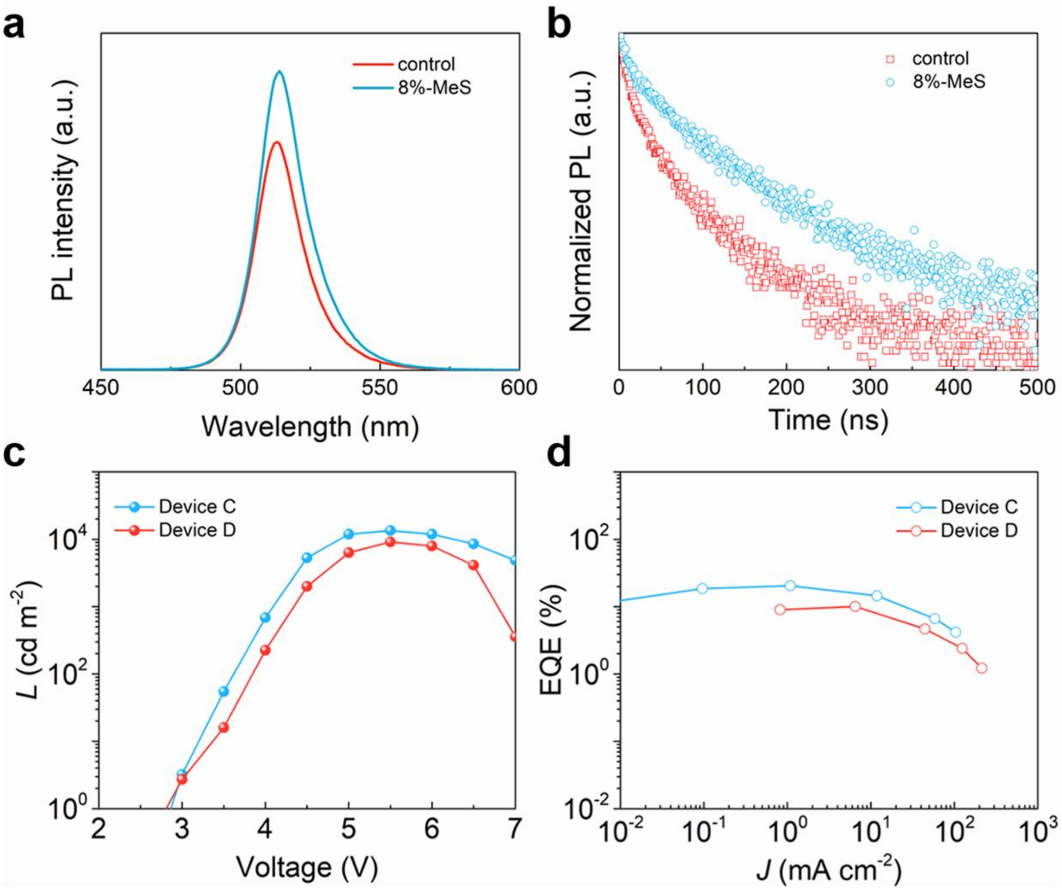

Supplementary Figure 12 | PL characteristics of perovskite films on TFB:PVK/PVP substrate and their EL performance. (a) Steady-state PL spectra and (b) TRPL decay of the control and MeS-treated perovskite film on TFB:PVK/PVP substrate excited at 365 nm. (c) *L*-voltage and (d) EQE-*J* curves of the Device C and Device D.

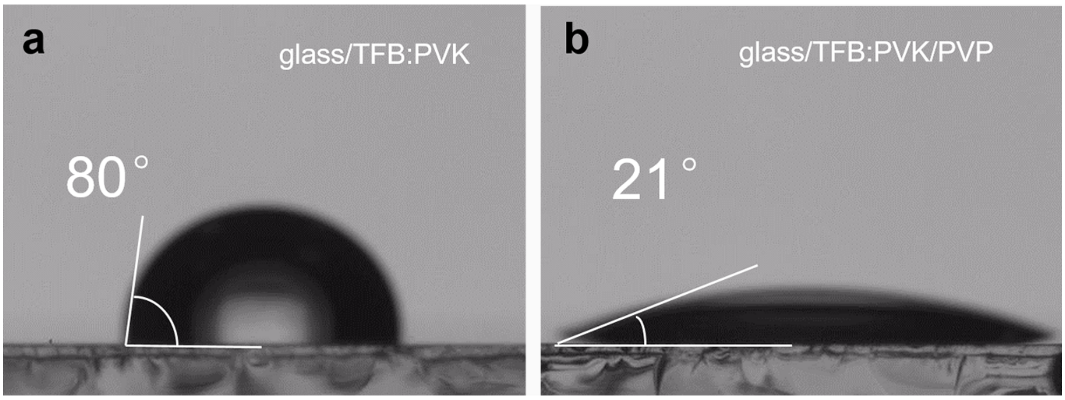

Supplementary Figure 13 | Contact angle measurements for perovskite precursor on different substrates. The contact angles of perovskite precursor on (a) glass/TFB:PVK and (b) glass/TFB:PVK/PVP substrates.

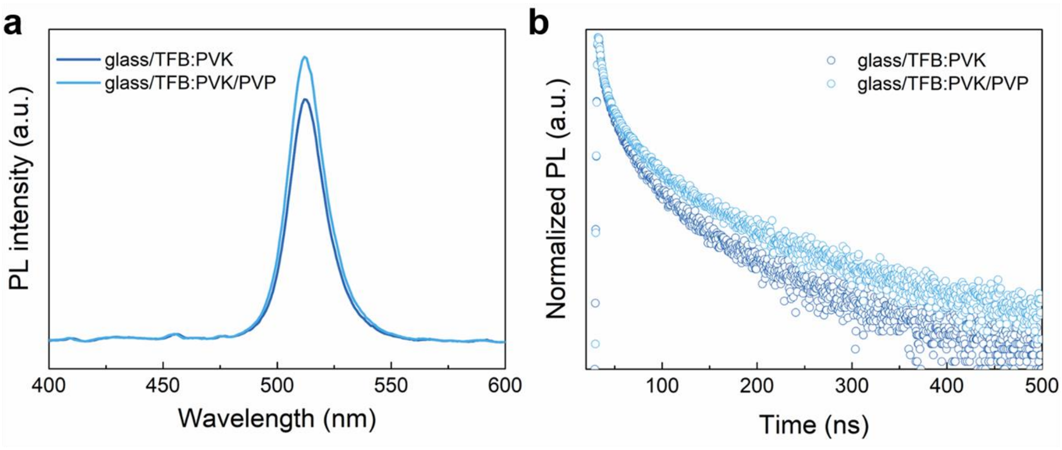

Supplementary Figure 14 | PL characteristics of perovskite films on different substrates.

(a) PL spectra and (b) TRPL decay curves of the 8%-MeS treated perovskite films on glass/TFB:PVK and glass/TFB:PVK/PVP substrates.

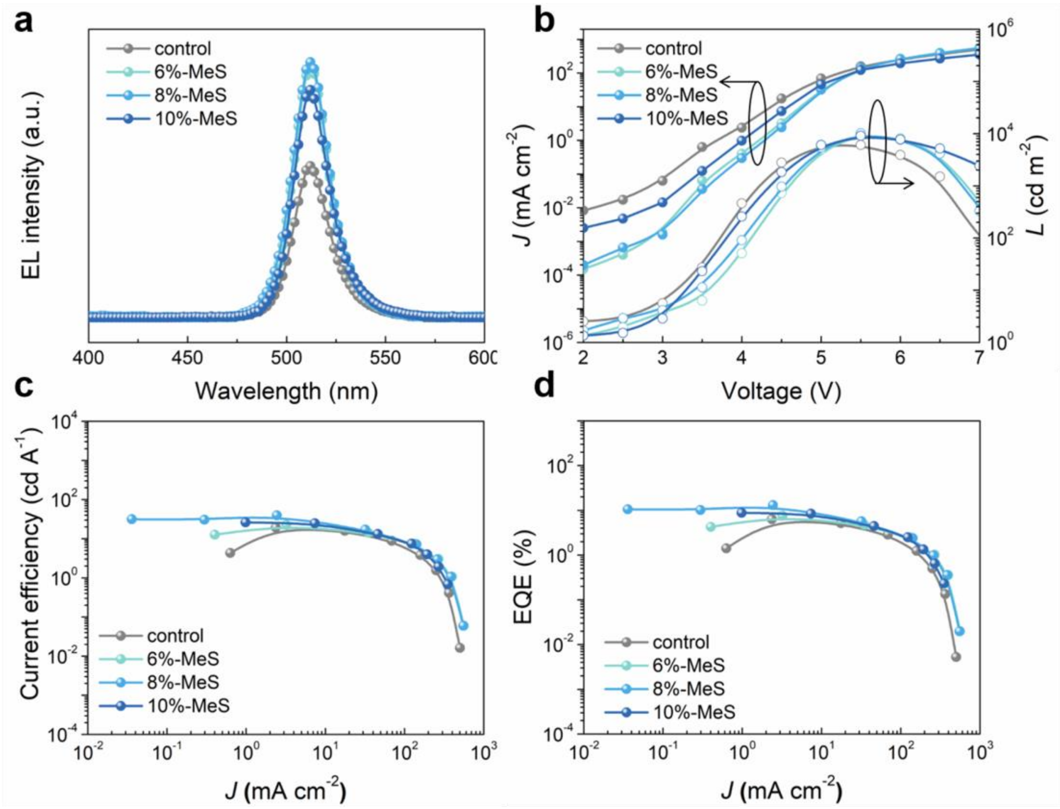

Supplementary Figure 15 | EL performance of MeS-treated PeLEDs. (a) EL spectra, (b) *J*-*V*-*L* characteristics, (c) CE-*J* and (d) EQE-*J* curves of PeLEDs based on *x*%-MeS treated perovskite films with a structure of ITO/TFB:PVK/Perovskite/TPBI/LiF/Al. Values of *x* are provided on the frames.

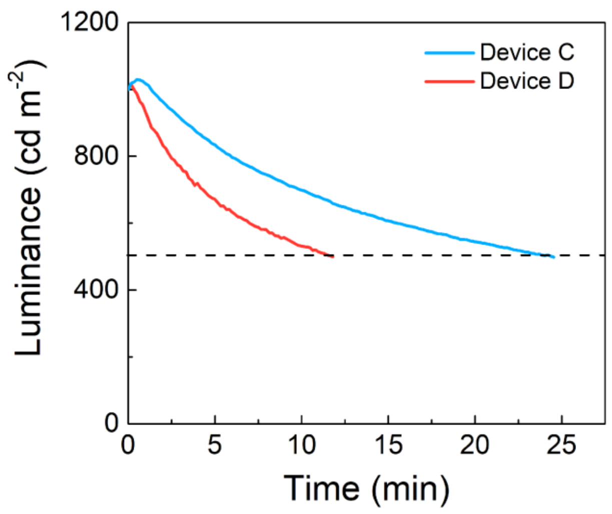

Supplementary Figure 16 | Operational lifetime of PeLEDs under constant driven current density. Device C shows a two-fold operational lifetime enhancement compared with the Device D.

Supplementary Table 1 | Formation time constants (τ_{et}) and first-order decays (τ_1) of each of the GSBs are shown in Fig. 2. The kinetics are fitted by a multiple-exponential function, $\Delta A(t) = a_1\exp(-t/\tau_1) + a_2\exp(-t/\tau_2) + a_3\exp(-t/\tau_3) - c_1\exp(-t/\tau_{et})$, where a_1, a_2, a_3 and c_1 are amplitudes; τ_1, τ_2 and τ_3 are decay time constants and τ_{et} is formation time constant. Only τ_1 and τ_{et} are listed due to the complex slow decay process of τ_2 and τ_3 such as energy slow transfer process, trap-assisted recombination, Auger recombination etc[2,3].

Samples	T	$GSB_{n=1}$ (404 nm)	$GSB_{n=2}$ (436 nm)	$GSB_{n=3}$ (465 nm)	$GSB_{n\geq4}$ (515 nm)
control	τ_1	0.14ps	0.33ps	0.97ps	-
	τ_{et}	0.08ps	0.10ps	0.13ps	1.20ps
8%-MeS	τ_1	-	0.11ps	0.26ps	-
	τ_{et}	-	0.09ps	0.12ps	0.90ps

Supplementary Table 2 | PL lifetime characteristics of *x*%-MeS treated perovskite films.

x [%]	τ_1 [ns]	A_1 [%]	τ_2 [ns]	A_2 [%]	τ_3 [ns]	A_3 [%]	τ_{avg} [ns]
control	5.36	11.58	22.46	36.30	87.31	52.11	76.64
8%	4.92	8.00	22.80	27.00	108.48	65.01	101.10

Supplementary Table 3 | Performance characteristics of PeLEDs based on *x*%-MeS treated perovskite films.

x [%]	Max. luminance [cd m^{-2}]	Max. CE [cd A^{-1}]	Max. EQE [%]
control	6094	19.42	6.37
6%	9791	22.49	7.52
8%	10140	39.37	13.14
10%	9117	26.23	8.76

Supplementary Table 4 | Performance summary for the quasi-2D PeLEDs reported in literature.

Reference	Emitter	EL color	Max. CE [cd A^{-1}]	Max. EQE [%]
Yang et al.[4]	$PEA_2(FAPbBr_3)_{n-1}PbBr_4$	Green	62.4	14.36
Lee et al.[5]	$BA_2FA_2Pb_3Br_{10}$	Green	62.4	14.60
Wu et al.[6]	$NMA_2Cs_{n-1}Pb_nBr_{3n+1}$	Green	46.80	14.90
Meng et al.[7]	$(PBA)(PA)A_{n-1}Pb_nX_{3n+1}$	Green	66.10	15.10
Yang et al.[8]	$PEA_2(FAPbBr_3)_2PbBr_4$	Green	-	15.40
Ban et al.[9]	$PEA_2Cs_{n-1}Pb_nBr_{3n+1}$	Green	49.10	15.50
Han et al.[10]	$PEA_2Cs_{n-1}Pb_nBr_{3n+1}$	Green	58.15	16.24
Liu et al.[11]	$PEA_2Cs_{n-1}Pb_nBr_{3n+1}$	Green	54.40	16.60
Zou et al.[12]	$NMA_2FA_{n-1}Pb_nI_{3n+1}$	NIR	-	12.70
He et al.[13]	$PBA_2Cs_{n-1}Pb_nI_{3n+1}$	Red	-	13.30
Zhao et al.[14]	$(NMA)_2(FA)_{n-1}Pb_nI_{3n+1}$	NIR	-	20.10
Li et al.[15]	$PEA_2Cs_{n-1}Pb_n(Cl_{0.9}Br_{2.1})_{3n+1}$	Blue	-	5.70
Wang et al.[16]	$PEA_2Cs_{n-1}Pb_nBr_{3n+1}$	Blue	-	6.30
Ren et al.[3]	$PEA_2Cs_{n-1}Pb_n(Cl_xBr_{1-x})_{3n+1}$	Blue	-	7.51
Liu et al.[17]	$PBABr_y(Cs_{0.7}FA_{0.3}PbBr_3)$	Blue	12.00	9.50
Wang et al.[18]	$CsPbBr_3$:PEACl	Blue	-	11.00
Chu et al.[19]	$PEA_2(Cs_{1-x}EA_xPbBr_3)_2PbBr_4$	Blue	-	12.10
**Our work**	$\mathbf{BA_2Cs_{n-1}Pb_nBr_{3n+1}}$	**Green**	**63.00**	**20.50**

Supplementary References

1. You, M., et al. Improving efficiency and stability in quasi-2D perovskite light-emitting diodes by a multifunctional LiF interlayer. *ACS Appl. Mater. Interfaces* **12,** 43018-43023 (2020).
2. Jiang, Y., et al. Spectra stable blue perovskite light-emitting diodes. *Nat. Commun.* **10,** 1868 (2019).
3. Ren, Z., et al. Simultaneous low-order phase suppression and defect passivation for efficient and stable blue light-emitting diodes. *ACS Energy Lett.* **5,** 2569-2579 (2020).
4. Yang, X. et al. Efficient green light-emitting diodes based on quasi-two-dimensional composition and phase engineered perovskite with surface passivation. *Nat. Commun.* **9,** 570 (2018).
5. Lee, S. et al. Control of interface defects for efficient and stable quasi-2D perovskite light-emitting diodes using nickel oxide hole injection layer. *Adv. Sci.* **5,** 1801350 (2018).
6. Wu, T. et al. Nanoplatelet modulation in 2D/3D perovskite targeting efficient light-emitting diodes. *Nanoscale* **10,** 19322-19329 (2018).
7. Meng, F. et al. Co-interlayer engineering toward efficient green quasi-two-dimensional perovskite light-emitting diodes. *Adv. Funct. Mater.* **30,** 1910167 (2020).
8. Yang, X. et al. Effects of organic cations on the structure and performance of quasi-two-dimensional perovskite-based light-emitting diodes. *J Phys. Chem. Lett.* **10,** 2892-2897 (2019).
9. Ban, M. et al. Solution-processed perovskite light emitting diodes with efficiency exceeding 15% through additive-controlled nanostructure tailoring. *Nat. Commun.* **9,** 3892 (2018).
10. Han, B. et al. Novel lewis base cyclam self-passivation of perovskites without an anti-solvent process for efficient light-emitting diodes. *ACS Appl. Mater.*

Interfaces **12,** 14224-14232 (2020).

11. Liu, Y. et al. In-situ passivation perovskite targeting efficient light-emitting diodes via spontaneously formed silica network. *Nano Energy* **78,** 105134 (2020).
12. Zou, W. et al. Minimizing efficiency roll-off in high-brightness perovskite light-emitting diodes. *Nat. Commun.* **9,** 608 (2018).
13. He, Z. et al. High-efficiency red light-emitting diodes based on multiple quantum wells of phenylbutylammonium-cesium lead iodide perovskites. *ACS Photonics* **6,** 587-594 (2019).
14. Zhao, B. et al. High-efficiency perovskite-polymer bulk heterostructure light-emitting diodes. *Nature Photon.* **12,** 783-789 (2018).
15. Li, Z. et al. Modulation of recombination zone position for quasi-two-dimensional blue perovskite light-emitting diodes with efficiency exceeding 5%. *Nat. Commun.* **10,** 1027 (2019).
16. Wang, Y.-K. et al. Chelating-agent-assisted control of $CsPbBr_3$ quantum well growth enables stable blue perovskite emitters. *Nat Commun.* **11,** 3674 (2020).
17. Liu, Y. et al. Efficient blue light-emitting diodes based on quantum-confined bromide perovskite nanostructures. *Nature Photon.* **13,** 760-764 (2019).
18. Wang, Q. et al. Efficient sky-blue perovskite light-emitting diodes via photoluminescence enhancement. *Nat. Commun.* **10,** 5633 (2019).
19. Chu, Z. et al. Large cation ethylammonium incorporated perovskite for efficient and spectra stable blue light-emitting diodes. *Nat. Commun.* **11,** 4165 (2020).
